# Energy Efficient Pico Cell Range Expansion and Density Joint Optimization for Heterogeneous Networks with eICIC

**DOI:** 10.3390/s18030762

**Published:** 2018-03-02

**Authors:** Yanzan Sun, Wenqing Xia, Shunqing Zhang, Yating Wu, Tao Wang, Yong Fang

**Affiliations:** Shanghai Institute for Advanced Communication and Data Science, Key laboratory of Specialty Fiber Optics and Optical Access Networks, Joint International Research Laboratory of Specialty Fiber Optics and Advanced Communication, Shanghai University, Shanghai 200072, China; yanzansun@shu.edu.cn (Y.S.); wenqingxia_1993@163.com (W.X.); edwards-2105@163.com (S.Z.); twang@shu.edu.cn (T.W.); yfang@shu.edu.cn (Y.F.)

**Keywords:** HetNets, eICIC, energy efficiency, stochastic geometry

## Abstract

Heterogeneous networks, constituted by conventional macro cells and overlaying pico cells, have been deemed a promising paradigm to support the deluge of data traffic with higher spectral efficiency and Energy Efficiency (EE). In order to deploy pico cells in reality, the density of Pico Base Stations (PBSs) and the pico Cell Range Expansion (CRE) are two important factors for the network spectral efficiency as well as EE improvement. However, associated with the range and density evolution, the inter-tier interference within the heterogeneous architecture will be challenging, and the time domain Enhanced Inter-cell Interference Coordination (eICIC) technique becomes necessary. Aiming to improve the network EE, the above factors are jointly considered in this paper. More specifically, we first derive the closed-form expression of the network EE as a function of the density of PBSs and pico CRE bias based on stochastic geometry theory, followed by a linear search algorithm to optimize the pico CRE bias and PBS density, respectively. Moreover, in order to realize the pico CRE bias and PBS density joint optimization, a heuristic algorithm is proposed to achieve the network EE maximization. Numerical simulations show that our proposed pico CRE bias and PBS density joint optimization algorithm can improve the network EE significantly with low computational complexity.

## 1. Introduction

With years of data traffic exponential growth in wireless networks, energy costs and their contribution to global carbon dioxide emission are emerging as major concerns, which is a severe problem for cellular networks [1]. To meet the traffic exponential growth, the network capacity improvement is constrained by the limited spectral resources and severe energy consumption. How to improve the spectral efficiency while reducing the network energy consumption becomes of high importance for the wireless networks. Therefore, the network Energy Efficiency (EE) that considers both spectral efficiency and energy consumption has been valued not only as an important network performance indicator for modern wireless networks, but also for the operational expenditure reduction and sustainable development [2]. Heterogeneous networks (HetNets) consisting of a conventional macro cell deployment and overlaying pico cells have been investigated in the 3rd Generation Partnership Project (3GPP) Long Term Evolution (LTE)-Advanced as a promising paradigm shift to support the deluge of data traffic with higher spectral efficiency and EE [3,4,5,6]. With pico cells deployment in HetNets, wireless links to end users become shorter, which turns out to improve the link quality in terms of spectrum efficiency as well as the network EE [7]. It is estimated that HetNets will contribute 56-fold enhancement to the 1000-fold increase in traffic demand [8]. Consequently, HetNets is emerging as one of the core features for 5G cellular networks.

### 1.1. Motivation

In HetNets with Macro cell Base Stations (MBSs) and Pico cell Base Stations (PBSs), Cell Range Expansion (CRE) scheme is proposed to adjust the coverage of PBS and balance the load between MBSs and PBSs by adding a positive bias to their measured Reference Signal Received Power (RSRP) during cell association [9,10]. The pico edge User Equipment (UE), however, will suffer significant interference from MBSs, even causing the outage of control signal. As a result, the downlink interference mitigation needs to be performed even for control channels, which becomes a fundamental requirement for the network EE improvement.

Another feature called time domain Enhanced Inter-Cell Interference Coordination (eICIC) has been adopted as a baseline method for control channel interference mitigation in LTE-Advanced [11]. In eICIC schemes, each MBS remains silent for certain periods, termed as Almost Blank Subframe (ABS), over which PBS can schedule CRE area UEs with reduced interference [12]. On the basis of eICIC ABS technology, obtaining the optimal pico CRE bias will directly affect the number of UEs served by PBSs and eventually contribute to the network EE improvement. This is also one of the key issues to realize 5G green cellular networks [13,14].

Furthermore, to meet 1000 times wireless traffic volume increment in the near future, massive PBSs deployment will be necessary and the related EE problems will become severe due to the following reasons. First, the dense deployment of PBSs causes additional inter-tier and intra-tier interference, which is difficult to characterize. Second, most PBSs are typically deployed to satisfy the peak traffic volume, while the highly dynamic wireless traffic may result in low EE if massive PBSs are still awakened during low traffic periods such as midnight [15].

### 1.2. Related Works

Existing literature mainly focused on the pico CRE bias, ABS power and other parameters’ optimization for the network capacity maximization or link reliability improvement in HetNets [5,9,16,17], and recent works began to shift to the network EE optimization with the consideration of inter-tier data channel interference coordination and mitigation [18,19,20,21,22,23], including spectrum allocation [18,19], power control [20,21,22], and cognitive sensing based on the inter-cell co-channel downlink interference coordination [23]. In [24], the authors maximized the EE of pico cells in HetNets by means of some non-convex methods. To move one step further, a few works have tried to optimize the network EE considering pico CRE with eICIC technology [14,15,25]. For example, in [25], an adaptive ABS ratio configuration scheme was investigated to meet the network EE. In [14], the optimal transmission power of each MBS in the protected bands was developed by using Differential Evolution (DE) theory to enhance the system throughput and EE. In [15], the frequency allocation among cells in HetNets with interference coordination and user association for energy saving was optimized by adjusting the cell selection bias.

Another factor to improve the network EE is through PBSs density optimization [3,26,27,28]. In [26], a density threshold of small cells in ultra-dense cellular networks was investigated considering the backhaul network capacity and EE. In [27], the authors came up with an approximation algorithm to solve the intractable user association problem by controlling the PBS density dynamically. Furthermore, [3] proved that not only did the density of PBS have a notable impact on the network EE, but the density of MBSs could also affect the network EE. The authors of [28] optimized the PBS density and MBS density together through traffic-aware sleeping strategies to enhance the network EE. The authors of [29] optimized the BS transmit power and BS density jointly based on stochastic geometry theory. [30] investigated the distribution of transmit power for uplink UEs and the energy efficiency of uplink transmission in HetNets. In addition, PBSs density can be optimized by combining with other technologies to further improve the network EE. For example, Multiple-Input Multiple-Output (MIMO) technology and Coordinated Multipoint (CoMP) transmission were investigated to improve the network EE in [31,32,33,34,35,36,37]. Particularly, the authors of [34] proposed a low-complexity algorithm to figure out the minimum power consumption problem based on MIMO technology combining with small BSs’ deployment strategy. Reference [37] studied the Energy-Spectral Efficiency (ESE) benefiting from the joint optimization of CoMP transmission and Base Station (BS) density.

From the above, most existing works either researched the impacts of PBS density on the network EE, or studied the eICIC parameters (like Pico CRE bias, ABS power, etc.) influence on the network EE. However, few works jointly considered the PBS density and eICIC parameters effect on the network EE. By jointly considering the pico CRE bias and PBS density with eICIC technology, the total number of UEs offloaded from macro cells can be optimized with inter-tier interference coordinated and eventually the network EE can be improved. Hence, this paper analyzes the possible improvement of the network EE by jointly considering the PBS density and pico CRE bias with eICIC technology. The main contributions of this paper include two parts: (1) we first analytically derived the closed-form expression of the network EE as a function of the density of PBS and pico CRE bias by using stochastic geometry theory; (2) based on the derived network EE function, we proposed a low computational complexity PBS density and pico CRE bias joint optimization algorithm to achieve the globally optimal network EE.

The rest of this paper is organized as follows. The network model is described in Section 2. The analysis model is described in Section 3, where the closed-form expressions of the average achievable rate of users, the network power consumption and the network EE are derived. A low complexity joint PBS density and CRE bias tuning algorithm to maximize the network EE is proposed in Section 4. Numerical results and discussions are presented in Section 5. Concluding remarks are given in Section 6.

## 2. Network Model

Due to the randomness of the BSs deployment in HetNets, especially the PBSs, the traditional hexagonal cellular model cannot reflect the network deployment in reality precisely. Therefore, the authors of [38,39] proposed a tractable analytical model for homogeneous cellular network and HetNets, respectively, based on statistic geometry theory, where the location distribution of BSs is modeled as spatial Poisson Point process (PPP). Then, the closed-form expression of the network performance such as average rate, Signal to Interference plus Noise Ratio (SINR) coverage, rate coverage, etc., can be obtained to analyze the network performance conveniently [10,17,39,40,41,42]. Therefore, several authors studied the network EE optimization using stochastic geometry theory for HetNets [3,26,43,44], which is also adopted to model the network in this paper.

**Figure 1:**
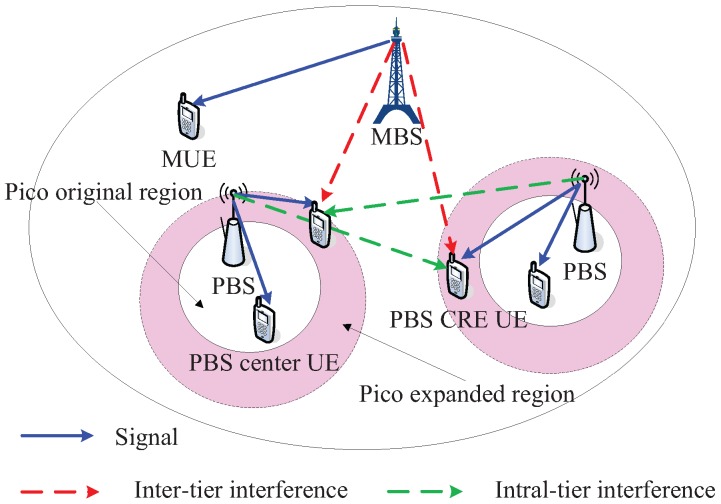
The network scenario.

A two-tier HetNets consisting of MBSs with higher transmission power and PBSs with lower transmission power is considered, as shown in Figure 1. Let $k\in \left\{1,2\right\}$ denote the tier index. Without any loss of generality, let the MBSs be tier 1 and the PBSs constitute tier 2. We assume that the MBSs and PBSs are spatially located according to a homogeneous PPP $\Phi_{m}$ and $\Phi_{p}$ with density $\lambda_{m}$ and $\lambda_{p}$, respectively in the Euclidean plane. The UEs are also distributed according to a different and independent homogeneous PPP $\Phi_{u}$ with density $\lambda_{u}$.

To mitigate the downlink interference from MBS to pico CRE users, the ABS scheme is adopted in MBS. Therefore, all the subframes are divided into two types of subframes for MBS, i.e., the ABS subframes and the Non-ABS subframes in time domain, respectively. We denote θ to be the ABS ratio, i.e., the proportion between the amount of ABS subframes and the number of the entire subframes. The transmission power of MBS in ABS and Non-ABS subframes are 0w and $P_{m}$, respectively. Note that, in an ABS subframe, PBSs still suffer Cell-specific Reference Symbol (CRS) interference from MBS, which is transmitted at regular intervals by MBS. Therefore, we further assume that CRS interference cancellation is employed by PBS for analysis simplicity. The transmission power of PBS is denoted as $P_{p}$.

We consider a cell association based on the maximum biased RSRP, where a UE is associated with the strongest BS in terms of the biased received RSRP at the user. In this paper, the association bias for MBS is assumed to be unity ($B_{m}=0$ dB) and that for PBS is pico CRE bias depicted as $B_{p}$. Without loss of generality, $B_{p}$ is preferably set to be larger than 0 dB; then, the coverage area of PBS can be expanded and the load of MBS can be better offloaded.

According to the cell association scheme, all of the UEs can be divided into three different types as shown in Figure 1: the type of MBS UEs contain the users connected to the MBSs, the type of PBS CRE UEs correspond to the users located in the expanded region of the PBSs (i.e., the user receiving a higher RSRP from the nearest MBS than that from the nearest PBS) and the type of PBS center UEs comprise the users distributed in the original coverage of PBSs (i.e., the user receiving a higher RSRP from the nearest PBS than that from the nearest MBS).

Based on the different subframe types and user types, the UEs scheduling can be executed as: each MBS remains silent on ABS subframes, over which PBSs can schedule PBS CRE UEs with reduced interference; the Non-ABS subframes will be assigned to the MBS UEs and the corresponding subframes for pico are allocated to the PBS center UEs.

Without loss of generality, we conduct analysis on a typical UE at the origin. This is justified by Slivyak theorem, which states that there is no difference in property observed either at a point of the PPP or at an arbitrary point [16]. We adopt the index $l\in L=\left\{m_{u},p_{c},p_{e}\right\}$ to denote the indication of the above three types of UEs, respectively, where $m_{u}$ represents MBS UEs, $p_{c}$ denotes the PBS center UEs, and $p_{e}$ signifies PBS CRE UEs.

The received signal power of a typical UE *l* from a BS of the *k*th tier at a distance of $r_{l}$ can be represented as $P_{k}hr_{l}^{-\alpha }$, where $P_{k}$ is the transmission power of BS in the *k*th tier, the variable *h* denotes the channel fast fading gain, which is modeled as Rayleigh distributed with average unit power, i.e., $h∼\exp\left(1\right)$, the term α is the large scale path loss exponent, which is assumed to be the same in both of the two tiers for analysis simplicity. Thus, the SINR of a typical UE *l* according to its user type can be expressed as:

(1) $$\gamma_{l}=\left\{\begin{array}{c} \frac{P_{m}hr_{l}^{-\alpha }}{\sum_{k=1}^{2}I_{k,l}+\sigma^{2}}\;\mathrm{if}\;l=m_{u},\\ \frac{P_{p}hr_{l}^{-\alpha }}{\sum_{k=1}^{2}I_{k,l}+\sigma^{2}}\;\mathrm{if}\;l=p_{c},\\ \frac{P_{p}hr_{l}^{-\alpha }}{I_{2,l}+\sigma^{2}}\;\mathrm{if}\;l=p_{e}, \end{array}\right.$$

where $I_{k,l}$ denotes the interference from the *k*th tier to UE *l*.

We restrict that the PBS CRE UEs can only be scheduled by PBSs in the subframes that correspond to the MBS ABS subframes. Therefore, when $l\;=\;p_{e}$, the interference from MBSs, i.e., tier 1, can be omitted when the CRS interference cancellation is utilized in pico. This is why we just consider $I_{2,l}$, i.e., the intral-tier interference from PBS tier, in the denominator of the SINR expression when *l* = $p_{e}$.

## 3. Analytical Model

### 3.1. User Type Probability

We assume that the nearest distances from a typical UE to a PBS and a MBS are denoted by $r_{p}$ and $r_{m}$, respectively. Generally speaking, the user type of this typical UE can be defined according to the relationship between the biased received signal strength from its nearest MBS and PBS, respectively, as Equation (2) below:

(2) $$l=\left\{\begin{array}{c} m_{u},\mathrm{when}\;P_{m}hr_{m}^{-\alpha }>B_{p}P_{p}hr_{p}^{-\alpha },\\ p_{c},\mathrm{when}\;P_{p}hr_{p}^{-\alpha }>P_{m}hr_{m}^{-\alpha },\\ p_{e},\mathrm{when}\;P_{p}hr_{p}^{-\alpha }<P_{m}hr_{m}^{-\alpha }<B_{p}P_{p}hr_{p}^{-\alpha }. \end{array}\right.$$

**Lemma** **1.** *The probability of this typical UE belonging to the user type l can be defined as* $A_{l}=\mathrm{Prob}\left[l\in L\right]$, *which is given as below:*

(3) Al=λmλm+BpP^p22ααλp,whenl=mu,λpλp+P^m22ααλm,whenl=pc,λpλp+Bp−1P^m22ααλm−λpλp+P^m22ααλm,whenl=pe,

*where* P^p=PpPpPmPm, P^m=PmPmPpPp.

Proof: See Appendix A.

### 3.2. Distribution of Serving BS Distance

**Lemma** **2.** *Corresponding to the user type, the Probability Density Function (PDF) of the distance* $r_{l}$ *between a typical UE l and its serving BS can be expressed as Equation* (4), *respectively:*

(4) fmurl=2πrlλmAmuexp−πrl2λm+BpP^p22ααλp,fpcrl=2πrlλpApcexp−πrl2λp+P^m22ααλm,fperl=2πrlλpApeexp−πrl2λp+Bp−1P^m22ααλm−exp−πrl2λp+P^m22ααλm.

Proof: See Appendix B.

### 3.3. The Ratio of Almost Blank Subframe

We set the value of the ABS ratio θ to be the proportion between PBS CRE UE user type probability and the sum of the PBS CRE UE user type probability and the PBS center UE user type probability, as shown in Equation (5):

(5) θ=ApcApc+Ape=1−λp+Bp−1P^m22ααλmλp+P^m22ααλm.

### 3.4. Average Ergodic Rate

Assume that the network adopts full buffer traffic model and all the users in the coverage of a BS share the entire frequency resource equally. Thus, the mean achievable downlink data rate of a typical UE *l* can be represented as follows:

(6) $$R_{l}=\frac{W_{l}}{E\left[N_{l}\right]}E\left[\log_{2}\left(1+\gamma_{l}\right)\right],$$

where $N_{l}$ is the mean load in a Voronoi cell and the expectation of $N_{l}$ is ENl=AlAlρkρk+1. When $l\;=\;m_{u}$, then ρk=ρ1=λmλmλuλu. When $l\in \left\{p_{c},\rho_{e}\right\}$, then ρk=ρ2=λpλpλuλu. $W_{l}$ is the time-frequency resource that is allocated to user *l* and its value depends on the user type of user *l*. Specifically, when $l\;=\;p_{e}$, then $W_{l}=\;\theta W$ and when $l\in \left\{m_{u},p_{c}\right\}$, then $W_{l}=\left(1-\theta \right)W$.

Based on the analysis above, we get Lemma 3 in the following:

**Lemma** **3.** *The average achievable downlink rate of a typical UE l can be further deduced as:*

(7) $$R_{l}=\frac{2\pi \lambda_{l}W_{l}}{A_{l}N_{l}}\int_{0}^{\infty }\int_{0}^{\infty }\exp\left(-\varphi_{l}-\pi {r_{l}}^{2}C_{l}\right)f_{l}\left(r_{l}\right)dr_{l}dt,$$

*where* $\tau =2^{t}-1$, $\varphi_{l}=-\tau \sigma^{2}{r_{l}}^{2}P_{k}^{-1}$, $C_{l}=\left\{\begin{array}{c} \lambda_{m}Z\left(\tau ,\alpha ,1\right)+\lambda_{p}Z\left(\tau ,\alpha ,B_{p}\right),\mathrm{when}\;l\;=\;m_{u}\\ \lambda_{m}{\hat{P}}_{m}Z\left(\tau ,\alpha ,1\right)+\lambda_{p}Z\left(\tau ,\alpha ,1\right),\mathrm{when}\;l\;=\;p_{c}\\ \lambda_{p}Z\left(\tau ,\alpha ,1\right),\mathrm{when}\;\;l\;=\;p_{e} \end{array}\right.$, *where* Zτ,α,β=τ22αα∫ββττ22αα∞11+xαα22dx.

Proof: See Appendix C.

**Corollary** **1.** With noise ignored, and setting the large scale path loss exponent α=4, the average achievable downlink rate of a typical UE *l* can be simplified, respectively, according to its user type as follows:

(8) $$\begin{array}{c} R_{m_{u}}=\frac{\left(1-\theta \right)W}{A_{m_{u}}N_{m_{u}}}\int_{0}^{\infty }\frac{1}{Q\left(\tau ,4,1\right)+\frac{\lambda_{p}}{\lambda_{m}}Q\left({\hat{P}}_{p}\tau ,4,B_{p}{\hat{P}}_{p}\right)}dt,\\ R_{p_{c}}=\frac{\left(1-\theta \right)W}{A_{p}N_{p_{c}}}\int_{0}^{\infty }\frac{1}{Q\left({\hat{P}}_{m}\tau ,4,{\hat{P}}_{m}\right)+\frac{\lambda_{m}}{\lambda_{p}}Q\left(\tau ,4,1\right)}dt,\\ R_{p_{e}}=\frac{\theta W}{A_{p_{e}}N_{p_{e}}}\int_{0}^{\infty }\frac{1}{Q\left(\tau ,4,1\right)+{\left(B_{p}^{-1}{\hat{P}}_{m}\right)}^{1/2}\frac{\lambda_{m}}{\lambda_{p}}}\\ \begin{array}{cc} & -\frac{1}{Q\left(\tau ,4,1\right)+{\left({\hat{P}}_{m}\right)}^{1/2}\frac{\lambda_{m}}{\lambda_{p}}}dt, \end{array} \end{array}$$

where Qτ,4,x=x+τtan−1ττxx.

Proof: when α=4, $\sigma^{2}=0$, and let $\varphi_{l}=0$ in Equation (7), then we can get Zτ,α,β=τ∫ββττ∞11+x2dx=τarctanττββ. Combining with Equation (7), we will obtain the desired results.

### 3.5. BS Power Consumption

Generally, BS consumes two types of power: static power consumption and transmit power consumption [3]. Then, for the *k*th tier, a BS power consumption can be given as follows:

(9) $$P_{k}=P_{k,s}+\xi_{k}P_{k,t},$$

where $P_{k,s}$ is the static power consumption of a BS in the *k*th tier, which is caused by signal processing, battery backup, as well as site cooling, and is independent with the BS transmit power. $P_{k,t}$ is the transmit power of a BS for data transmission in the *k*th tier, and $\xi_{k}$ is the load-dependent power consumption coefficient of a BS in the *k*th tier.

Note that the transmit powers of each MBS in ABS subframe and Non-ABS subframe are different. Therefore, the network power consumption in ABS subframe and Non-ABS subframe will not be the same. Thus, we decompose the network power consumption into two parts: the network power consumption in ABS subframe $P_{abs}$ and the network power consumption in Non-ABS subframe $P_{\text{non\_abs}}$. In particular, the transmit power consumption of MBS for data transmission in ABS subframe is assumed to be zero due to its silence in the ABS subframe. Combined with Equation (9) and the density of MBS and PBS, $P_{abs}$ and $P_{\text{non\_abs}}$ can be obtained in the following, respectively:

(10) $$\begin{array}{c} P_{abs}=\lambda_{m}P_{1,s}+\lambda_{p}P_{2,s}+A_{p_{e}}\lambda_{u}P_{2,t}\\ \;\;\;=\lambda_{m}P_{m,s}+\lambda_{p}P_{p,s}+A_{p_{e}}\lambda_{u}P_{p,t}, \end{array}$$

(11) $$\begin{array}{c} P_{non\_abs}=\lambda_{m}P_{1,s}+A_{m_{u}}\lambda_{u}P_{1,t}+\lambda_{p}P_{2,s}+A_{p_{c}}\lambda_{u}P_{2,t}\\ \;\;\;=\lambda_{m}P_{m,s}+A_{m_{u}}\lambda_{u}P_{m,t}+\lambda_{p}P_{p,s}+A_{p_{c}}\lambda_{u}P_{p,t}. \end{array}$$

Considering the ABS ratio θ, the network total power consumption can be derived as:

(12) $$P_{total}=\theta P_{abs}+\left(1-\theta \right)P_{\text{non\_abs}}.$$

### 3.6. Network Energy Efficiency

The network EE can be defined as the ratio of the effective network throughput over the network total power consumption:

(13) $$EE=\frac{R_{total}}{P_{total}}=\frac{R_{total}}{\theta P_{abs}+\left(1-\theta \right)P_{\text{non\_abs}}}.$$

For convenience of derivation, we set α=4, $\sigma^{2}\;=0$. Then, combining Equations (8), (9)–(13), the expression of the network EE is obtained as follows:

(14) EE=RtotalPtotal=RmuAmu+RpcApc+RpeApeλuθPabs+1−θPnon_abs=λuPtotal∫0∞1−θW1−θWNmuNmuQτ,4,1+ρp,mQP^pτ,4,BpP^p+1−θW1−θWNpcNpcQP^mτ,4,P^m+ρp,m−1Qτ,4,1−θWθWNpeNpeQτ,4,1+P^m1/2ρp,m−1+θWθWNpeNpeQτ,4,1+Bp−1P^m1/2ρp,m−1dt,

where λuλuPtotalPtotal=λuλuλmPm,s+λpPp,s+θApePp,tλuλmPm,s+λpPp,s+θApePp,tλu+λu1−θAmPm,t+ApPp,t), ρp,m=λpλpλmλm.

## 4. Joint Parameters Optimization

Referring to Equation (14), the network EE is determined by $\rho_{p,m}$, i.e., the ratio between PBS density $\lambda_{p}$ and MBS density $\lambda_{m}$, UE density $\lambda_{u}$, MBS transmission power $P_{m}$, PBS transmission power $P_{p}$, ABS ratio θ and pico CRE bias $B_{p}$ together. Fortunately, the MBS transmission power $P_{m}$ and PBS transmission power $P_{p}$ are usually set to be constant. The MBS density changes slightly, hence we can also set the MBS density $\lambda_{m}$ in Equation (14) to be a constant value. In addition, the ABS ratio can be calculated according to Equation (5). Based on the analysis above, the network EE in Equation (14) can be maximized by $\rho_{p,m}$ (i.e., PBS density $\lambda_{p}$ due to the fact that MBS density $\lambda_{m}$ changes slightly) and pico CRE bias $B_{p}$ optimization with different UE densities, i.e., network load.

However, the network EE function is nonlinear with $\rho_{p,m}$ and $B_{p}$, which is a difficult task to solve the optimal PBS density and the optimal CRE bias at the same time to maximize the network EE. An alternative would be fixing one variable and solving for the other one. Note that the value ranges of $\rho_{p,m}$ and $B_{p}$ are limited , which make it possible to seek out the optimal $\rho_{p,m}$ and $B_{p},$ respectively, through a linear search algorithm by fixing one of these two variables. Then, a heuristic algorithm is proposed to optimize these two variables jointly. By defining $0<B_{p}\leq 25\;\mathrm{dB}$ and $0<\rho_{p,m}\leq 30$, the objective function can be expressed as

(15) $$\begin{array}{c} \underset{B_{p},\lambda_{p}}{\arg\max EE}=\frac{\left(R_{m_{u}}A_{m_{u}}+R_{p_{c}}A_{p_{c}}+R_{p_{e}}A_{p_{e}}\right)\lambda_{u}}{\theta P_{abs}+\left(1-\theta \right)P_{non\_abs}},\\ \begin{array}{cc} s.t. & 0<B_{p}\leq 25\;\mathrm{dB}, \end{array}\\ \;\;0<\rho_{p,m}\leq 30. \end{array}$$

### 4.1. Optimization of Pico CRE Bias

For the sake of getting the optimal CRE bias for network EE maximization, suppose that the ratio between PBS density and MBS density $\rho_{p,m}$ is a known quantity. Further assume that $\lambda_{u}$ is a constant value, which can be adjusted to represent different network loads. Then, the pico CRE bias optimization problem can be formulated as follows:

(16) $$\begin{array}{c} \underset{B_{p}}{B_{p}^{*}=\arg\max EE}\left(B_{p}\right)\left|{}_{\lambda_{p},\rho_{p,m}},\right.\\ \begin{array}{cc} s.t. & 0<B_{p}\leq 25\;\mathrm{dB}, \end{array}\\ \;\;\rho_{p,m}\;\mathrm{is}\;\mathrm{an}\;a\;\mathrm{arbitrary}\;\mathrm{constant}\;\mathrm{between}\;0\;\mathrm{and}\;30. \end{array}$$

Suppose that the variable step length of $B_{p}$ is 0.1. Thus, the optimal pico CRE bias for the network EE maximization can be obtained by means of a linear search algorithm, which is described in Algorithm 1.

**Algorithm 1:** CRE Bias Optimization (CBO) Algorithm. 1. **Initialization**: (1) Initialize the network scenario and the values $\lambda_{u}$, $\lambda_{m}$ and $\rho_{p,m}$, where $\rho_{p,m}\in \left(0,30\right]$. (2) Set the initial value of $B_{p}\;=\;0.1$. (3) Denote κ as the variable step length of $B_{p}$. Denote $EE^{*}$ as the optimal value of the network EE. Denote $B_{p}^{*}$ as the optimized CRE bias. Let $B_{p}^{*}=B_{p}$, $EE^{*}=EE\left(B_{p}\right)\left|{}_{\lambda_{p},\rho_{p,m}}\right.$ and κ=0.1. 2. **Calculate the optimal pico CRE bias** **while**
$B_{p}\leq 25$
**do** $B_{p}=B_{p}+\kappa$ $EE^{\prime }=EE\left(B_{p}\right)\left|{}_{\lambda_{p},\rho_{p,m}}\right.$ according to Equation (14) **if**
$EE^{\prime }>EE^{*}$
**then**  $B_{p}^{*}=B_{p}$, $EE^{*}=EE^{\prime }$ **end if** **end while**

### 4.2. Optimization of PBS Density

As the MBS density can be set as a constant, the PBS density optimization problem can be converted to $\rho_{p,m}$ optimization to maximize the network EE. After the optimized $\rho_{p,m}^{*}$ is obtained, then the optimal PBS density can be calculated by $\lambda_{p}^{*}=\lambda_{m}\rho_{p,m}^{*}$. Similarly, suppose that the PBS CRE bias $B_{p}$ is a known quantity. Further suppose that $\lambda_{u}$ is a constant value. Thus, the PBS density optimization problem can be formulated as follows:

(17) $$\begin{array}{c} \underset{\rho_{p,m}}{\rho_{p,m}^{*}=\arg\max EE}\left(\rho_{p,m}\right)\left|{}_{\lambda_{u},B_{p}},\right.\\ \begin{array}{cc} s.t. & 0<\rho_{p,m}\leq 30, \end{array}\\ \;\;B_{p}\;\mathrm{is}\;\mathrm{an}\;a\;\mathrm{arbitrary}\;\mathrm{constant}\;\mathrm{between}\;0\;\mathrm{and}\;25. \end{array}$$

Assume that the variable step length of $\rho_{p,m}$ is set to be 0.03. Thus, the optimal PBS density optimization for the network EE maximization can be also obtained by means of a linear search algorithm, which is shown in Algorithm 2.

**Algorithm 2:** PBS Density Optimization (PDO) Algorithm. 1. **Initialization**: (1) Initialize the network scenario and the values $\lambda_{u}$, $\lambda_{m}$ and $B_{p}$, where $B_{p}\in \left(0,25\right]$. (2) Set the initial value of $\rho_{p,m}=0.03$. (3) Denote σ as the variable step length of $\rho_{p,m}$. Denote $EE^{*}$ as the optimal value of the network EE. Denote $\rho_{p,m}^{*}$ as the optimized ratio between PBS density MBS density. Let $\rho_{p,m}^{*}=\rho_{p,m}$, $EE^{*}=EE\left(\rho_{p,m}\right)\left|{}_{\lambda_{u},B_{p}}\right.$ and σ=0.03. 2. **Calculate the optimal ratio of PBS density to MBS density** **while**
$\rho_{p,m}\leq 30$
**do** $\rho_{p,m}=\rho_{p,m}+\sigma$ $EE^{\prime }=EE\left(\rho_{p,m}\right)\left|{}_{\lambda_{u},B_{p}}\right.$ according to Equation (14) **if**
$EE^{\prime }>EE^{*}$
**then**  $\rho_{p,m}^{*}=\rho_{p,m}$, $EE^{*}=EE^{\prime }$ **end if** **end while** 3. **Obtain the optimal PBS density** $\lambda_{p}^{*}=\lambda_{m}\rho_{p,m}^{*}$

### 4.3. Joint Optimization of Pico CRE Bias and PBS Density

Based on the aforementioned parameter optimization algorithms, each parameter is just optimized with the other one being fixed, which in fact cannot reach these parameters’ global optimization due to the fact that these parameters are affected by each other. Therefore, combining the CBO Algorithm and PDO Algorithm, we further propose a heuristic pico CRE bias and PBS density joint optimization algorithm to address the objective Equation (15), which is as shown in Algorithm 3.

**Algorithm 3:** Joint Pico CRE Bias and PBS Density Optimization (JBPDO) Algorithm. 1. **Initialization**: (1) Initialize the network scenario and the values $\lambda_{u}$, $\lambda_{m}$, $B_{p}$ and $\rho_{p,m}$, where $B_{p}\in \left(0,25\right]$ and $\rho_{p,m}\in \left(0,30\right]$. (2) Let $EE^{*}=0$ represent the initial optimal value of the network EE. Initialize algorithm iteration number $N_{loop}=0$. Given a tolerance ε>0. (3) Denote $B_{p}^{*}$ as the optimized PBS CRE bias. Denote $\rho_{p,m}^{*}$ as the optimized ratio of PBS density to MBS density. Denote $\lambda_{p}^{*}$ as the optimized PBS density. 2. **Calculate the suboptimal CRE bias according to CBO Algorithm** $B_{p}^{\text{sub\_opt}}=\underset{B_{p}}{\arg\max EE}\left(B_{p}\right)\left|{}_{\lambda u},\rho_{p,m}\right.$ $B_{p}=B_{p}^{\text{sub\_opt}}$ 3. **Calculate the suboptimal ratio of PBS density to MBS density according to PDO Algorithm** $\underset{\rho_{p,m}}{\rho_{p,m}^{\text{sub\_opt}}=\arg\max EE}\left(\rho_{p,m}\right)\left|{}_{\lambda_{u},B_{p}^{\text{sub\_opt}}}\right.$ $\rho_{p,m}=\rho_{p,m}^{sub\_opt}$ $EE^{\text{sub\_opt}}=EE\left(B_{p}^{sub\_opt},\rho_{p,m}^{sub\_opt}\right)$
4. **Termination of the loop** **if**
$\left|EE^{\text{sub\_opt}}-EE^{*}\right|>\varepsilon$
**then** $EE^{*}=EE^{\text{sub\_opt}}$, $N_{loop}=N_{loop}+1$, go to step 2 **else** $B_{p}^{*}=B_{p}$, $\rho_{p,m}^{*}=\rho_{p,m}$ $\lambda_{p}^{*}=\lambda_{m}\rho_{p,m}^{*}$, $EE^{*}=EE^{\text{sub\_opt}}$ **end if**

## 5. Numerical Results and Analysis

A square of 1000m×1000m network coverage area is considered in the simulation. The deployment of PBSs and MBSs follows the PPP model and the typical user is placed in the origin. The detailed simulation parameters are summarized in Table 1. Monte Carlo simulation method is adopted to illustrate our proposed algorithms. In Monte Carlo simulation, the locations of MBSs, PBSs and UEs are modeled as spatial PPP for each time of the network realization, respectively. According to the network topology realized by spatial PPP model, the network EEs will be calculated for all the different combinations of pico CRE bias and PBS density values within their value ranges based on wireless link quality. Then, the maximum network EE and the optimal pico CRE bias and PBS density can be obtained by comparing all the calculated network EEs. By averaging 50 times of the network realizations, Monte Carlo simulation results can be obtained.

### 5.1. Performance Analysis for Pico CRE Bias Optimization

**Figure 2:**
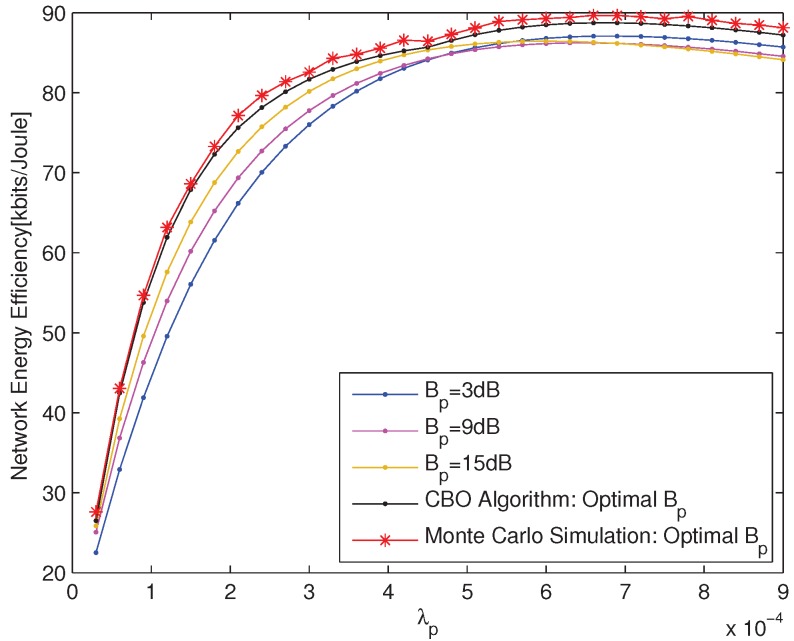
The network EE versus $\lambda_{p}$ with $\lambda_{u}=0.0018$.

**Figure 3:**
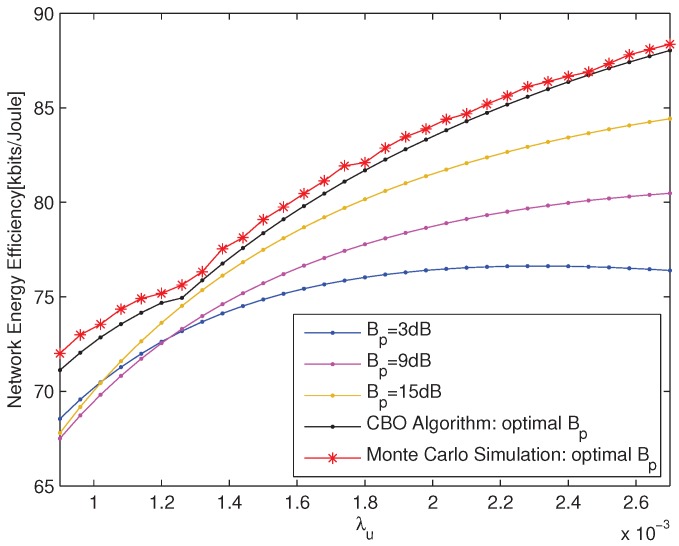
The network EE versus $\lambda_{u}$ with $\lambda_{p}=0.0006$.

The performances of the optimal pico CRE bias obtained according to CBO Algorithm are compared with those of the static pico CRE bias and Monte Carlo simulation, which are shown in Figure 2 and Figure 3 from different aspects, respectively. The network EE for the static pico CRE bias is calculated according to Equation (14) and the static pico CRE bias is set to be 3 dB, 9 dB and 15 dB, respectively. The Monte Carlo simulation results show the maximum network EE within the value range of PBS density.

The network EE versus $\lambda_{p}$ with UE density $\lambda_{u}=0.0018$ is shown in Figure 2. As shown in Figure 2, the performances of CBO Algorithm always outperform those of static pico CRE bias and agreed with those of Monte Carlo simulation very well, which illustrate on the one side the accuracy of our derived closed form of network EE and on the other side the effectiveness of our proposed CBO Algorithm. Furthermore, simulation results show that no matter what the value of pico CRE bias is, all the curves of network EE keep increasing with PBS density increasing at the initial stage, and then tend to fall down slightly with PBS density further increasing. This indicates that increasing the PBS density deployment can improve the network EE significantly with a certain UE density. Nonetheless, when the PBS density achieves a certain level, further increasing of it will not only cause a lot of interference to users, but also give rise to more power consumption, and eventually deteriorate the network EE.

The network EE versus $\lambda_{u}$ with fixed PBS density $\lambda_{p}=0.0006$ is depicted in Figure 3, which shows that the network energy efficiencies of CBO Algorithm are always better than those of static pico CRE bias and capture the results of Monte Carlo simulation very well. This is due to the fact that the CBO Algorithm can optimize the number of UEs offloaded from macro cell on the basis of the network EE. In addition, with the increase of UE density, the performance gap between CBO Algorithm and static pico CRE bias increase accordingly, which further signifies the importance of pico CRE bias optimization for the heavy network load scenario.

### 5.2. Performance Analysis for PBS Density Optimization

**Figure 4:**
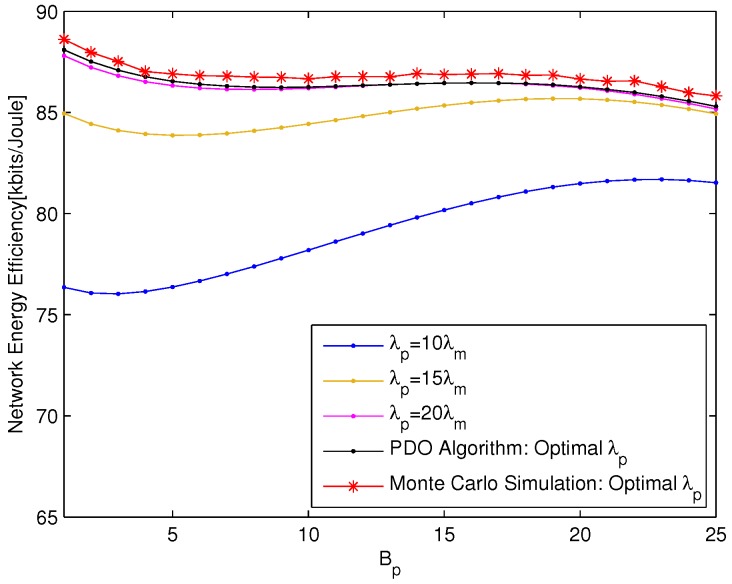
The network EE versus $B_{p}$ with $\lambda_{u}=0.0018$.

**Figure 5:**
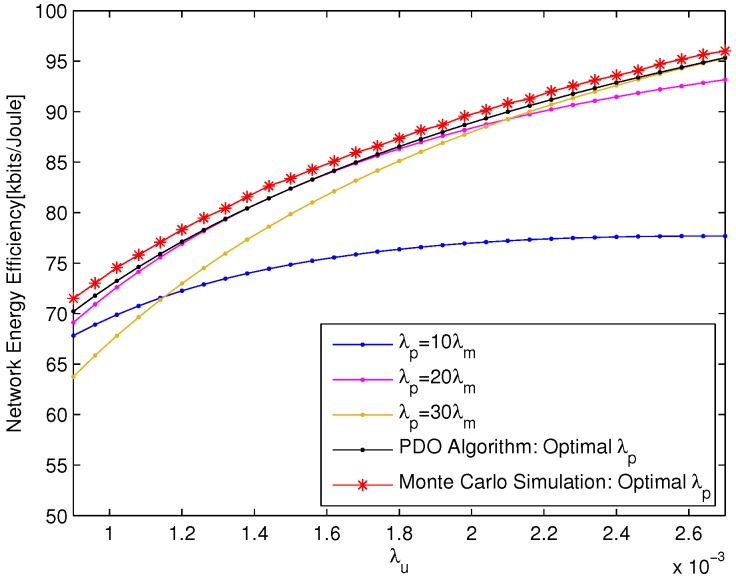
The network EE versus $\lambda_{u}$ with $B_{p}=5\;\mathrm{dB}$.

The performances of the optimal PBS density obtained according to PDO Algorithm are compared with those of the static PBS density (represented by the static ratio of PBS density to MBS density) and Monte Carlo simulation, which are presented in Figure 4 and Figure 5 from different aspects, respectively. The network EE for the static ratio of PBS density to MBS density is obtained according to Equation (14) and the static ratio $\rho_{p,m}$ is set to be 10, 15 and 20, respectively. The Monte Carlo simulation results show the maximum network EE within the value range of PBS density and are averaged over 50 times of network realizations.

The relationship between the network EE and $B_{p}$ with UE density $\lambda_{u}=0.0018$ is depicted in Figure 4. The network EE versus $\lambda_{u}$ with pico CRE bias $B_{p}=5\;\mathrm{dB}$ is described in Figure 5. Both of the simulation results show that the performances of PDO Algorithm outperform those of the static PBS densities and fit those of Monte Carlo simulation precisely. Figure 4 also shows that the network EE is nonlinear with $B_{p}$, which signifies the difficulty of $B_{p}$ optimization by closed-form solution. As shown in Figure 5, the curves of static PBS densities cross with each other under different UE densities, which indicates that the PBS density should be carefully adjusted according to the network load fluctuation. This is why the performance of our proposed PDO Algorithm can always outperform those of static PBS density scheme.

### 5.3. Performance Analysis for Joint Optimization of CRE Bias and PBS Density

**Figure 6:**
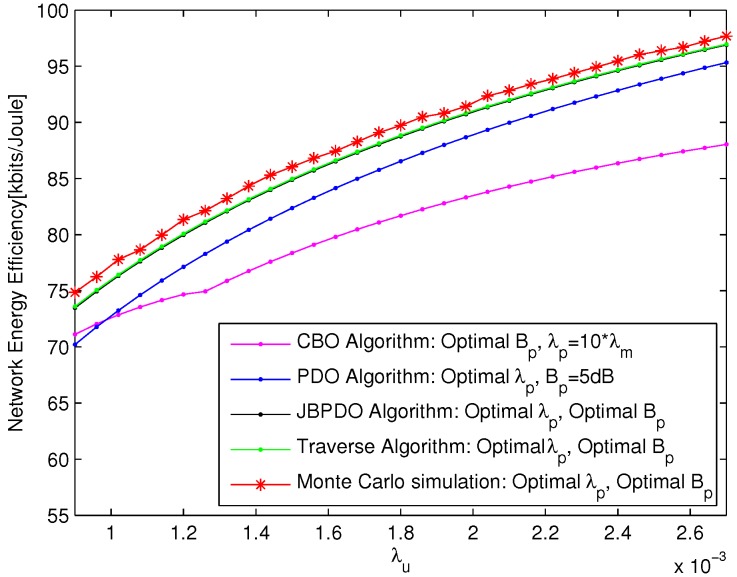
The network EE versus $\lambda_{u}$ with different optimization algorithms.

The network EE performance of JBPDO Algorithm are compared with those of a CBO Algorithm with fixed $\lambda_{p}=20\lambda_{m}$, PDO Algorithm with fixed $B_{p}=5$ dBm, Traverse Algorithm and Monte Carlo simulation in Figure 6. Traverse Algorithm simulation results are obtained by traversing all the values of pico CRE bias and PBS density to find optimal CRE bias and PBS density based on Equation (14) to maximize the network EE. The Monte Carlo simulation results show the maximum network EE within the value ranges of PBS density and pico CRE bias together and are averaged over 50 times of network realizations. Clearly, due to the fact that the JBPDO Algorithm can jointly optimize pico CRE bias and PBS density together to achieve these two parameters global optimization, the number of UEs offloaded from macro cells can be further optimized and eventually the JBPDO Algorithm can further enhance the network EE compared with CBO Algorithm and PDO Algorithm. Monte Carlo simulation results once again illustrate the accuracy and effectiveness of our proposed JBPDO Algorithm.

**Figure 7:**
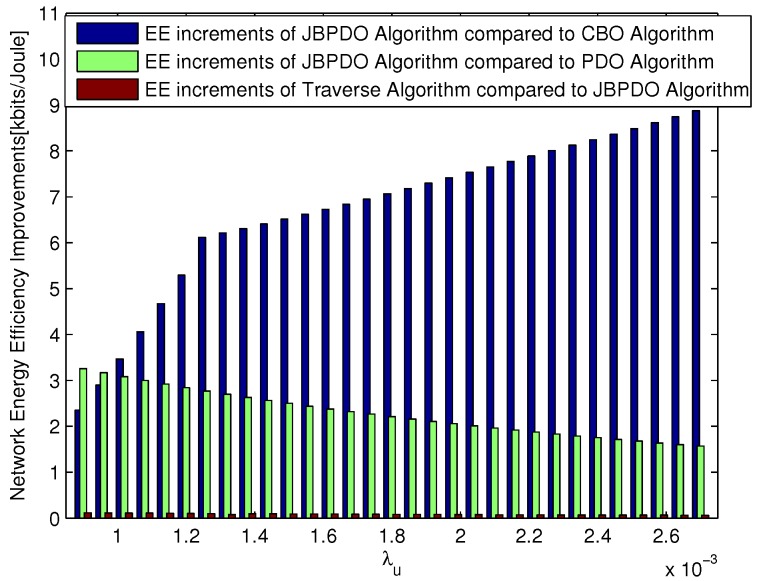
The network EE performance improvement comparison between different algorithms.

The network EE increments of JBPDO Algorithm compared with CBO Algorithm and PDO Algorithm are depicted in Figure 7 under different user densities. As shown in Figure 7, due to the fact that JBPDO Algorithm can realize pico CRE bias and PBS density joint optimization, the performance of JBPDO Algorithm can always outperform CBO Algorithm and PDO Algorithm obviously with different user densities. The network EE performance gap is also given in Figure 7 between our proposed JBPDO Algorithm and Traverse Algorithm, which can also realize pico CRE bias and PBS density joint optimization with higher computational complexity. Simulation results show that the network EE performance of our proposed JBPDO Algorithm is just slightly worse than that of Traverse Algorithm.

**Figure 8:**
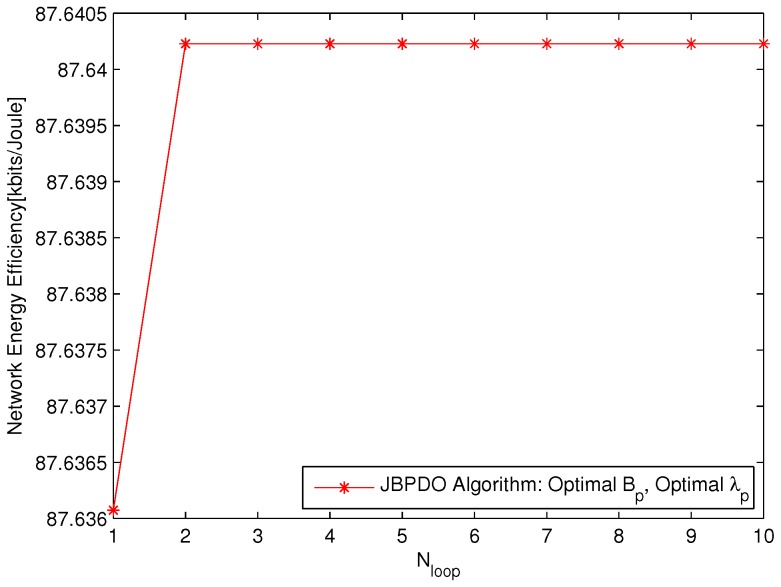
The network EE versus iteration times $N_{l}oop$ of JBPDO Algorithm.

Referring to Figure 6 and Figure 7 together, the performance of Traverse Algorithm outperforms JBPDO Algorithm slightly. However, the computational complexity of JBPDO Algorithm is much lower than that of Traverse Algorithm. The computational complexity of Traverse Algorithm will be $O\left(n_{p,cre}\times n_{p,density}\right)$ and that of JBPDO Algorithm will be $O\left[\left(n_{p,cre}+n_{p,density}\right)\times N_{loop}\right]$. The convergence of JBPDO Algorithm is simulated in Figure 8, which is averaged with 30 different UE densities. It is seen from Figure 8 that JBPDO Algorithm can converge after two iterations. Hence, although the network EE performance of JBPDO Algorithm is slightly smaller than that of Traverse Algorithm, the computational complexity of JBPDO is much lower than that of Traverse Algorithm.

## 6. Conclusions

In this paper, we analyze the network EE for a two-tier HetNets consisting of MBSs and PBSs by means of stochastic geometry theory, where CRE implemented on PBS and ABS based on an eICIC scheme is adopted by MBS for downlink interference mitigation to PBS CRE UEs. We first derive the closed-form expression of the network EE. Then, a linear search algorithm is adopted to optimize the pico CRE bias and PBS density, respectively. Finally, a heuristic based algorithm is proposed to optimize the pico CRE bias and PBS density jointly to achieve the network EE maximization. Extensive simulation results show the accuracy of our theory deduction and the effectiveness of our proposed optimization algorithms for the network EE optimization with reduced complexity.

## Figures and Tables

**Table 1:** Network scenario parameters.

Parameters	Value
Carrier frequency *f*	2 GHz
Path loss exponent α	4
Path Loss *L*	$L=10\log\left(L_{0}\right)+\alpha 10\log\left(r_{l}\right)$, where L0=4πf4πfcc2, $c=3\times 10^{8}\;m/s$
MBS transmit power $P_{m}$ or $P_{m,t}$	43 dBm or 20 W
PBS transmit power $P_{p}$ or $P_{p,t}$	30 dBm or 1 W
Bandwidth *W*	10 MHz
MBS static power $P_{m,s}$	800 W
PBS static power $P_{p,s}$	130 W
MBS density $\lambda_{m}$	0.00003

## Appendix A

**Proof** **of** **Lemma** **1.** Considering a typical user, it is at a distance *r* away from its closest BS in the *k*th tier. Then, there is no BS closer than *r* in the *k*th tier. Due to the fact that the void probability of 2D PPP in an area of Ψ is $e^{-\lambda_{k}\Psi }$, we can obtain

(A1) $$P\left(r_{k}>r\right)=P\left(no\;BS\;closer\;than\;r_{k}\right)=e^{-\lambda_{k}\pi r^{2}},$$

where $r_{k}$ denotes the distance of this typical user to the closest BS in the *k*th tier, and $\lambda_{k}$ is the BS density of *k*th tier. Then, we can get the cumulative distribution function (CDF) of $r_{k}$ as follows:

(A2) $$F_{r_{k}}\left(r\right)=1-\exp\left(-\lambda_{k}\pi r^{2}\right).$$

Hence, the probability density function (PDF) of the distance $r_{k}$ is

(A3) $$f_{r_{k}}\left(r\right)=2\pi \lambda_{k}r\exp\left(-\pi \lambda_{k}r^{2}\right).$$

Referring to the user type formular expressiones in Equation (2), we deduce the probability of a similar expression $Ar_{2}<r_{1}<Br_{2}$ as:

(A4) $$\begin{array}{c} P\left(Ar_{2}<r_{1}<Br_{2}\right)=P\left(Ar_{2}<r_{1}<Br_{2},r_{2}\right)\\ =\int_{0}^{\infty }P\left(Ar_{2}<r_{1}<Br_{2}\right)f_{r_{2}}\left(r\right)dr\\ =\int_{0}^{\infty }\left[\int_{Ar}^{Br}2\pi \lambda_{1}r\exp\left(-\pi \lambda_{1}r^{2}\right)dr\right]f_{r_{2}}\left(r\right)dr\\ =\int_{0}^{\infty }\left[\exp\left(-\lambda_{1}\pi A^{2}r^{2}\right)-\exp\left(-\lambda_{1}\pi B^{2}r^{2}\right)\right]\\ \begin{array}{cc} & \cdot \end{array}2\pi \lambda_{2}r\exp\left(-\pi \lambda_{2}r^{2}\right)dr\\ =\frac{\lambda_{2}}{\lambda_{2}+A^{2}\lambda_{1}}-\frac{\lambda_{2}}{\lambda_{2}+B^{2}\lambda_{1}}, \end{array}$$

where *A* and *B* denote the arbitrary value coefficients and the subscript 1 and 2 denote the tier 1 and tier 2, respectively. In the same way, we can get the expressions of $A_{m_{u}}$, $A_{p_{c}}$ and $A_{p_{e}}$ combining Equations (2) and (A4).  ☐

## Appendix B

**Proof** **of** **Lemma** **2.** To get the PDF of the distance between a typical user and its serving BS, the user type of this typical UE has to be identified firstly. Then, this issue can be converted to solving a conditional probability problem, which can be solved by Bayes rule. Assuming that the distance between the typical user and its serving BS is $r_{l}$, then the PDF of it according to its user type can be expressed as:

(A5) $$\begin{array}{c} f_{m_{u}}\left(r_{l}\right)=f_{m_{u}}\left(r_{m}\left|P_{m}hr_{m}^{-\alpha }>B_{p}P_{p}hr_{p}^{-\alpha }\right.\right),\\ f_{p_{c}}\left(r_{l}\right)=f_{p_{c}}\left(r_{p}\left|P_{p}hr_{p}^{-\alpha }>P_{m}hr_{m}^{-\alpha }\right.\right),\\ f_{p_{e}}\left(r_{l}\right)=f_{p_{e}}\left(r_{p}\left|P_{p}hr_{p}^{-\alpha }<P_{m}hr_{m}^{-\alpha }<B_{p}P_{p}hr_{p}^{-\alpha }\right.\right), \end{array}$$

where $f_{m_{u}}\left(r_{l}\right)$, $f_{p_{c}}\left(r_{l}\right)$ and $f_{p_{e}}\left(r_{l}\right)$ have similar expressions. If we can deduce the results of $f_{p_{e}}\left(r_{l}\right)$, then $f_{m_{u}}\left(r_{l}\right)$ and $f_{p_{c}}\left(r_{l}\right)$ can be obtained in the same way. Referring to the expression of $f_{p_{e}}\left(r_{l}\right)$, we first calculate the following similar conditional probability:

(A6) $$P\left[r_{2}\left|Ar_{2}<r_{1}<Br_{2}\right.\right]=\frac{P\left[r_{2}<r,Ar_{2}<r_{1}<Br_{2}\right]}{P\left[Ar_{2}<r_{1}<Br_{2}\right],}$$

where the subscripts 1 and 2 denote tier 1 and tier 2 of HetNets, respectively, and *A* and *B* represent the arbitrary value coefficients. $P\left[Ar_{2}<r_{1}<Br_{2}\right]$ has been obtained in Equation (A4) and is independent of *r*. Therefore, the PDF of distance $r_{2}$ can be obtained by the derivation of the CDF function without considering $P\left[Ar_{2}<r_{1}<Br_{2}\right]$. Substituting *C* for $P\left[Ar_{2}<r_{1}<Br_{2}\right]$, the PDF of distance $r_{2}$ can be expressed as:

(A7) $$\begin{array}{c} f_{r_{2}}\left(r\right)=\frac{d}{dr}P\left[r_{2}\left|Ar_{2}<r_{1}<Br_{2}\right.\right]\\ =\frac{1}{C}\frac{d}{dr}P\left[r_{2}<r,Ar_{2}<r_{1}<Br_{2}\right]\\ =\frac{2\pi r\lambda_{2}}{C}\left\{\exp\left[-\pi r^{2}\left(\lambda_{2}+A\lambda_{1}\right)\right]\right.\\ \begin{array}{cc} & \left.-\exp\left[-\pi r^{2}\left(\lambda_{2}+B\lambda_{1}\right)\right]\right\}. \end{array} \end{array}$$

Similarly, we can also get the expression of $f_{m_{u}}\left(r_{l}\right)$, $f_{p_{c}}\left(r_{l}\right)$ and $f_{p_{e}}\left(r_{l}\right)$.  ☐

## Appendix C

**Proof** **of** **Lemma** **3.** On the basis of the expression of the SINR in Equation (1), $W_{l}$ and $N_{l}$ can be seen as constants. $f_{l}\left(r\right)$ can be obtained according to Equation (A7). Thus, we can deduce the expression of the average user rate of user type *l* as:

(A8) $$\begin{array}{c} R_{l}=\frac{W_{l}}{N_{l}}E\left[\log_{2}\left(1+SINR_{l}\right)\right]\\ =\frac{W_{l}}{N_{l}}\int_{0}^{\infty }E\left[\log_{2}\left(1+\frac{P_{l}hr_{l}^{-\alpha }}{\sum_{k=1}^{2}I_{l,k}+\sigma^{2}}\right)\right]f_{l}\left(r\right)dr \end{array}],$$

where

(A9) $$\begin{array}{c} E\left[\log_{2}\left(1+\frac{P_{l}hr_{l}^{-\alpha }}{\sum_{k=1}^{2}I_{l,k}+\sigma^{2}}\right)\right]\\ \overset{\left(a\right)}{=}\int_{0}^{\infty }\left[\log_{2}\left(1+\frac{P_{l}hr_{l}^{-\alpha }}{\sum_{k=1}^{2}I_{l,k}+\sigma^{2}}\right)>t\right]dt\\ =\int_{0}^{\infty }\left[h>\left(2^{t}-1\right)\left(\sum_{k=1}^{2}I_{l,k}+\sigma^{2}\right)r_{l}^{\alpha }P_{l}^{-1}\right]dt\\ \overset{\left(b\right)}{=}\int_{0}^{\infty }\exp\left[-\tau \sigma^{2}r_{l}^{\alpha }P_{l}^{-1}\right]\exp\left[-\tau \right.\left.I_{l,1}r_{l}^{\alpha }P_{l}^{-1}\right]\\ \begin{array}{cc} & \cdot exp\left[-\tau I_{l,2}r_{l}^{\alpha }P_{l}^{-1}\right]dt, \end{array} \end{array}$$

where (a) is derived according to $E\left(X\right)=\int_{0}^{\infty }P\left[X>x\right]dx$, (b) is obtained referring to $h∼\exp\left(\mu \right)$ and by setting $2^{t}-1=\tau$ and μ=1. Substituting the following Laplace transform Equation (A10) into Equation (A9), and further substituting Equation (A9) into Equation (A8), we can then obtain the expression of the average rate of user type *l* in the network, which is expressed in Equation (7):

(A10) Ll,kτrlαPl−1=exp−πλkPkPl22ααr2τ22αα∫βkβkττ22αα∞11+x22ααdx.

 ☐
